# Peroxiredoxin 2 is essential for maintaining cancer stem cell-like phenotype through activation of Hedgehog signaling pathway in colon cancer

**DOI:** 10.18632/oncotarget.13559

**Published:** 2016-11-24

**Authors:** Rong Wang, Jinlai Wei, Shouru Zhang, Xingye Wu, Jinbao Guo, Maoxi Liu, Kunli Du, Jun Xu, Linglong Peng, Zhenbing Lv, Wenxian You, Yongfu Xiong, Zhongxue Fu

**Affiliations:** ^1^ Department of Gastrointestinal Surgery, The First Affiliated Hospital, Chongqing Medical University, Chongqing 400016, China

**Keywords:** Prdx2, stemness, cancer stem cell, Hedgehog, colon cancer

## Abstract

Cancer stem cells (CSCs) are a key target for reducing tumor growth, metastasis, and recurrence. Redox status is a critical factor in the maintenance of CSCs, and the antioxidant enzyme Peroxiredoxin 2 (Prdx2) plays an important role in the development of colon cancer. Therefore, we investigated the contribution of Prdx2 to the maintenance of stemness of colon CSCs. Here, we used short-hairpin RNAs and a Prdx2-overexpression vector to determine the effects of Prdx2. We demonstrated that knockdown of Prdx2 reduced the self-renewal and sphere formation and resulted in increased 5-FU-induced apoptosis in human colon CSCs. Prdx2 overexpression induced reversion of the self-renewal and sphere formation. Furthermore, the effects of Prdx2 resulted in an altered expression of stemness associated with the Hh/Gli1 signaling pathway. Finally, knockdown of Prdx2 in CD133^+^ cells reduced the volume of xenograft tumors in BALB/c-nu mice. Taken together, colon CSCs overexpress Prdx2, which promotes their stem cell properties via the Hh/Gli1 signaling pathway. The results suggest that Prdx2 may be an effective therapeutic target for the elimination of CSCs in colorectal cancer.

## INTRODUCTION

Colorectal cancer is the fifth most common cancer and remains one of the leading causes of cancer-related deaths in China [1], emphasizing the need for a better understanding of the biology of colorectal cancer and for the development of new therapeutic strategies based on that biology. The limited effectiveness of the standard anti-cancer therapies against colorectal cancer has been attributed to the existence of the relatively rare, highly drug-resistant, and slowly proliferating population of tumor-driving cells termed cancer stem cells (CSCs) [2]. Therefore, the identification and characterization of CSC targets are critical for the design of novel targeted therapies to specifically suppress CSCs in colorectal cancer.

Several recent studies have reported that reactive oxygen species (ROS) are associated with the stemness of CSCs. Diehn *et al*. reported that low levels of ROS may be conducive to the maintenance of CSCs and to their resistance to ionizing radiation [3]. Similarly, Chen *et al*. found that ROS antagonists may help to maintain the stemness of hematopoietic stem cells [4]. Ye *et al*. reported that lung cancer stem cells (LCSCs) contained lower intracellular concentrations of ROS relative to non-LCSCs and that ROS generation increased during the differentiation of LCSCs [5]. Collectively, these studies indicate that redox status plays an important role in CSC maintenance.

Peroxiredoxins (Prdxs) define a superfamily of intracellular antioxidant enzymes in cells [6]. Prdxs have been reported to be differentially expressed in human pancreatic cancer [7], Burkitt lymphoma [8], lung carcinoma [9], colorectal carcinoma [10], prostate cancer [11], myeloma [12], and leukemia [13]. Prdx2, a typical 2-Cys peroxiredoxin, has higher expression levels in tumorous colon tissues compared with the corresponding normal non-tumor tissues [14], and Prdx2 knockdown inhibits cell growth and stimulates apoptosis [10]. However, as an important member of the ROS scavenging system, the contributions of Prdx2 to the maintenance of CSCs specifically involved in colon cancer have not been clearly defined.

The cell surface marker CD133 can be used to identify CSCs in colon cancer [15]. Our current study shows that the protein level of Prdx2 is markedly increased in CD133^+^ colon cancer cells compared with CD133^-^ cells. *In vitro* knockdown of Prdx2 reduced the CD133^+^ population and sphere formation in the SW620, HT29, and HCT116 colon cancer cell lines. Prdx2 depletion also caused a reduction in the mRNA and protein levels of CD44, CD133, and Nanog, as well as increased 5-fluorouracil (5-FU)-induced apoptosis. In our studies, we found a correlation between Prdx2 and CD133 at the protein expression level using immunohistochemical assays in human colon carcinoma tissues. In addition, Prdx2 depletion inhibited SMO and Gli1 expression in CD133^+^ cells. Furthermore, protein expression of SMO, Gli1, CD44, and CD133 was decreased in colon cancer cells in response to treatment with the SMO inhibitor cyclopamine. Finally, Prdx2 knockdown reduced the volume of xenograft tumors in BALB/c-nu mice. These data indicate that Prdx2 acts as a promoter of CSC properties in colon cancer via Hedgehog (Hh) signaling pathway.

## RESULTS

### Prdx2 is highly expressed in colon CSCs compared with non-CSCs

**Figure 1:**
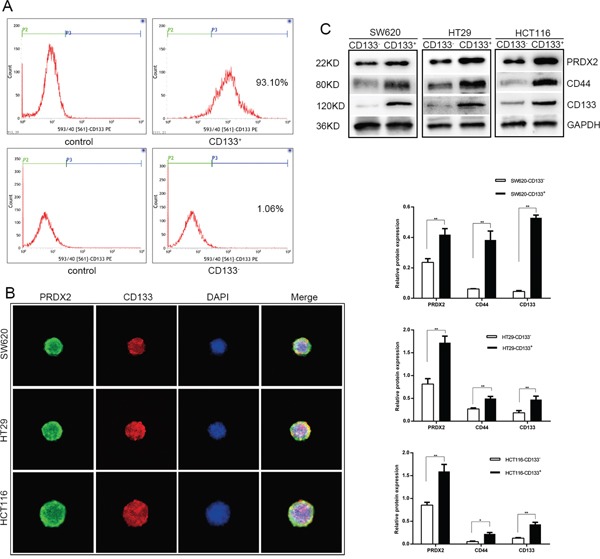
Prdx2 is up-regulated in CSCs **A**. CD133^+^ cells were sorted from human colon cancer cell line by magnetic activated cell sorting and the percentage of CD133^+^ population was assessed by flow cytometry. **B**. Prdx2 and CD133 protein expression in CSC spheres was visualized by immunofluorescent. **C**. Prdx2, CD44, and CD133 protein expression was confirmed by Western blot analysis of CD133^+^ and CD133^-^ cells isolated from SW620, HT29, and HCT-116 cell lines. *p< 0.05, **p< 0.01.

CD133 can be used to identify CSC from non-CSC. For further research in CSCs, CD133^+^ and CD133^-^ cells were sorted from human colon cancer cell lines, including SW620, HT29, and HCT116, by magnetic-activated cell sorting and identified by flow cytometry. The percentage of CD133-expressing cells in the CD133^+^ population reached 93.10%, while only 1.06% of the CD133^-^ cells (Figure 1A). To identify expression of Prdx2 and CD133 in CSC spheres, we acquired 3D spheres through serum-free culturing and detected protein expression with co-immunofluorescence (Figure 1B). To determine the effects of Prdx2 on the regulation of stemness, we analyzed the expression of Prdx2 as well as the cell surface markers CD133 and CD44 in the sorted CD133^+^ and CD133^-^ cells. We found that the expression of Prdx2 was significantly increased in the CD133^+^ population compared with the CD133^-^ population in all three cell lines (Figure 1C). These data shows that Prdx2 is overexpressed in CSCs from colon cancer compared with non-CSCs, which indicates Prdx2 may play an important role in CSC-correlated properties.

### Prdx2 knockdown results in a reduction of CSC-associated properties in colon cancer cells

**Figure 2:**
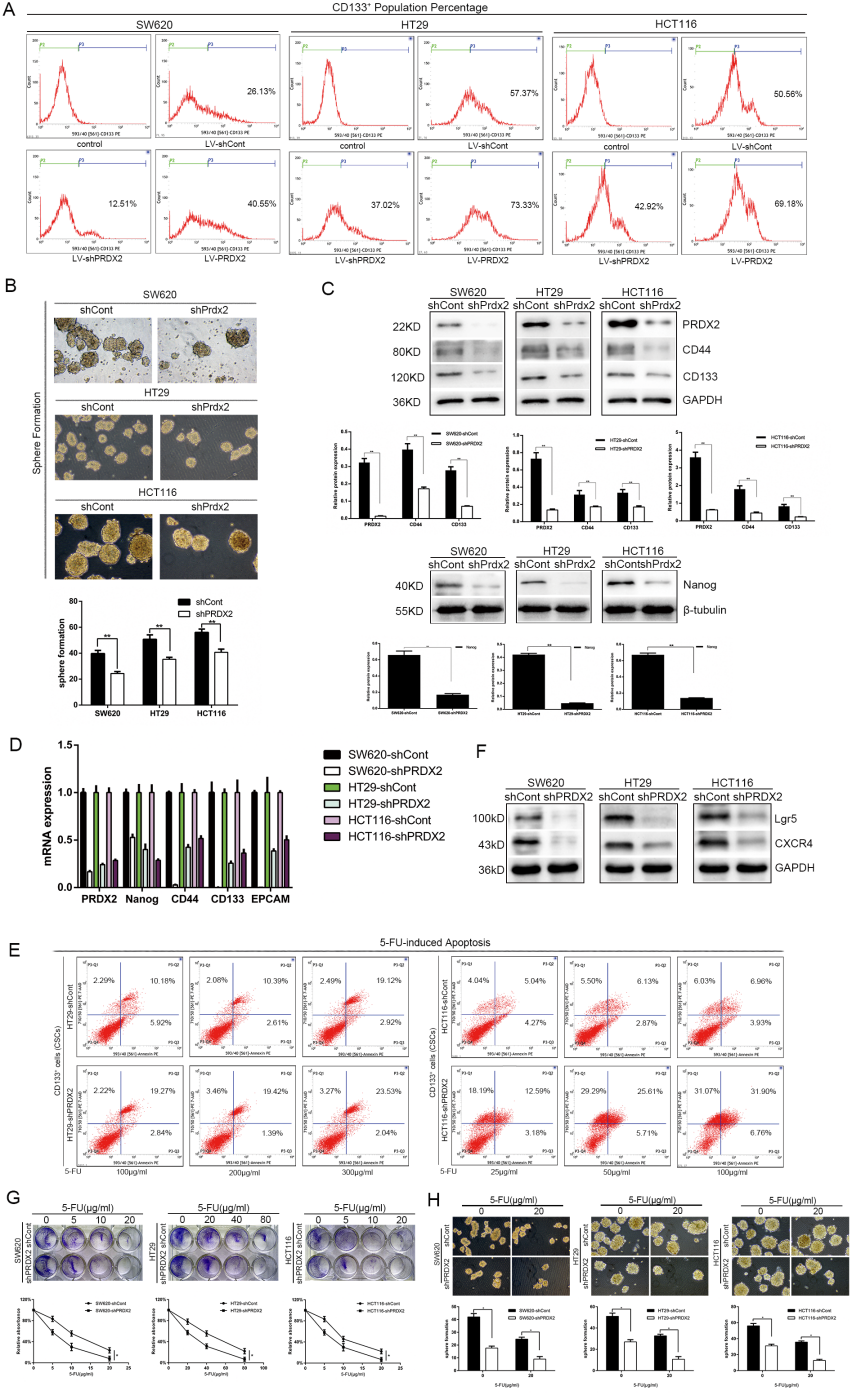
Prdx2 knockdown results in a reduction in stemness properties of colon cancer cells **A**. The percentage of CD133^+^ cells in stably-transduced shCont and shPrdx2 SW620, HT29, and HCT116 cell lines was analyzed by flow cytometry. **B**. Prdx2-depleted and control cells were subjected to sphere formation assays in ultra-low-attachment 6-well plates. The numbers of spheroids generated per 10,000 cells were counted 14 days after seeding. *p< 0.05, **p< 0.01. **C**. The protein levels of Prdx2, CD44, CD133, and Nanog in stably-transduced shCont and shPrdx2 SW620, HT29, and HCT116 cell lines were detected by Western blot analysis. *p< 0.05, **p< 0.01. **D**. The mRNA levels of Prdx2, CD44, CD133, EPCAM and Nanog in stably-transduced shCont and shPrdx2 SW620, HT29, and HCT116 cell lines were detected by quantitative polymerase chain reaction. **E**. CD133^+^ cells sorted from HT29 and HCT116 cells with and without Prdx2 depletion were treated with 5-fluorouracil for 48 h, and apoptosis levels were then detected by flow cytometry. **F**. The protein levels of Lgr5, and CXCR4 in stably-transduced shCont and shPrdx2 SW620, HT29, and HCT116 cell lines were detected by Western blot analysis. **G**. Lv-shPRDX2 and Lv-shCont transfected cells were treated with the indicated concentrations of 5-FU for 8 days and subjected to clonogenic cell survival assay. Quantitation of clonogenic cells from three independent experiments is shown. *p< 0.05. **H**. Lv-shPRDX2 and Lv-shCont transfected cells were treated with the indicated concentrations of 5-FU for 14 days and subjected to clonogenic spheres survival assay. Quantitation of clonogenic spheres from three independent experiments is shown. *p< 0.05.

To determine the role of Prdx2 in the modulation of CSC-correlated properties, Prdx2 levels were depleted by Prdx2-shRNA-LV transduction in the SW620, HT29, and HCT116 human colon cancer cell lines. Interestingly, we observed a significant reduction in the CD133^+^ population in the shPrdx2-transduced cells by flow cytometry compared with the negative control (shCont-transduced) cells (Figure 2A). Sphere formation assays revealed that the number of sphere colonies was also reduced by more than 10% in the Prdx2-depleted cells compared with the negative controls (Figure 2B). Quantitative reverse transcription-polymerase chain reaction (RT-PCR) and Western blot analysis of Prdx2, CD44, CD133, Lgr5, CXCR4 and Nanog revealed that Prdx2-depleted cells had lower levels of expression of all of these proteins (Figure 2C, 2D and 2F). Furthermore, treatment of the CD133^+^ cells sorted from the shPrdx2- and shCont-transduced cells with 5-FU for 48 h yielded a greater percentage of apoptotic cells in the Prdx2-depleted cells (Figure 2E). 5-FU treatment also inhibited cell proliferation and spheres formation, which was more significant in the Prdx2-depleted cells (Figure 2G and 2H). These findings show the significant effects of Prdx2 knockdown in the reduction of CSC-correlated properties.

### Prdx2 promotes the CSC-associated properties of colon cancer cells

**Figure 3:**
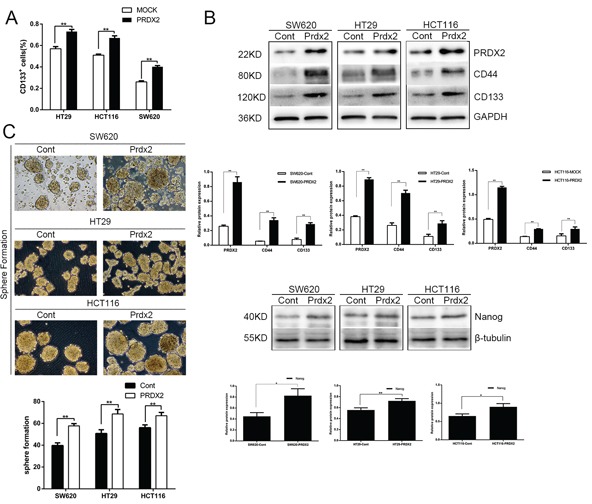
Prdx2 promotes the stemness and survival of colon cancer stem cells **A**. The percentage of CD133^+^ cells in Prdx2-overexpressing and control colon cancer cells was determined by flow cytometry. *p< 0.05, **p< 0.01. **B**. The increase in CD44, CD133, and Nanog protein expression in response to overexpression of Prdx2 in SW620, HT29, and HCT116 cells was assessed by Western blot analysis. *p< 0.05, **p< 0.01. **C**. Prdx2-overexpressed and control cells were subjected to sphere formation assays in ultra-low-attachment 6-well plates. The number of spheroids generated per 10,000 cells was counted 14 days after seeding. *p< 0.05, **p< 0.01.

To further define the role of Prdx2 in the modulation of CSC-associated properties, we overexpressed Prdx2 with Prdx2-GFP-LV transduction in the SW620, HT29, and HCT116 human colon cancer cell lines. We found that overexpression of Prdx2 significantly increased the percentage of CD133^+^ cells in all three colon cancer cell lines (Figure 2A and 3A). In addition, we observed a higher level of CD44, CD133, and Nanog protein expression in the Prdx2-overexpressing cells compared with the negative control cells (Figure 3B). Furthermore, Prdx2 overexpression also enhanced sphere formation in all three cell lines (Figure 3C).

### Prdx2 is associated with CD133 in colon carcinoma

**Figure 4:**
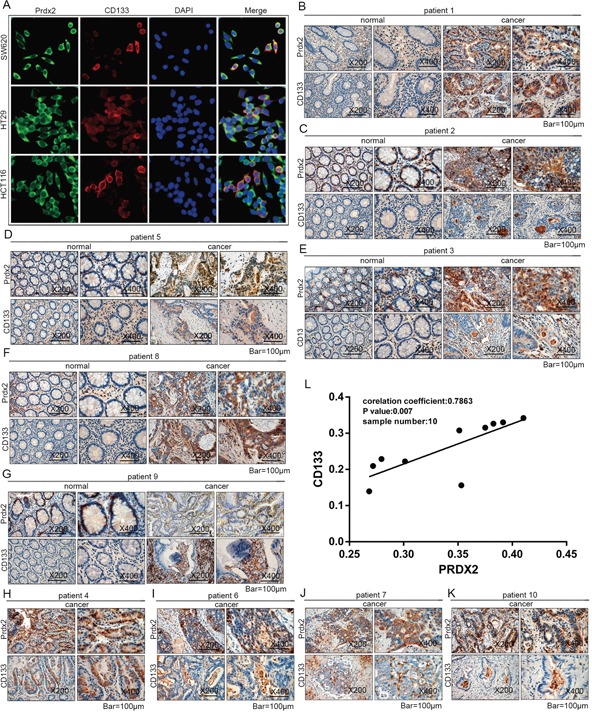
Prdx2 is associated with CD133 in colon carcinoma **A**. Prdx2 and CD133 protein expression in colon cancer cells (SW620, HT29, and HCT-116) was visualized by immunofluorescent. **B-K**. Protein expression of Prdx2, CD44, and CD133 in human colon carcinoma tissues and adjacent normal tissues from 10 patients was observed using an immunohistochemical assay. **L**. Integral Optical Density (IOD) of Prdx2 and CD133 protein expression in colon adenocarcinoma tissues from 10 patients was analyzed. The corresponding Pearson correlation coefficients and *P* values are shown.

To identify expression of Prdx2 and CD133 in colon cancer cells, co-immunofluorescence was applied in human colon cancer cells lines. We found Prdx2 expression in cell membrane and cytoplasm and CD133 expression in cell membrane (Figure 4A). Then, we detected Prdx2 and CD133 protein levels in human colon carcinoma samples from 10 patients (Table 1) with immunohistochemical assay and found higher expression in colon carcinoma tissues compared with adjacent normal tissues (Figure 4B-4K). To clarify the correlation of Prdx2 and CSCs, we assessed protein levels of Prdx2 and CD133 in colon carcinoma tissues by measuring Integrated Optical Density (IOD). A significant positive correlation (correlation coefficient = 0.7863, *P* < 0.05) was observed between Prdx2 and CD133 expression levels in colon carcinoma tissues from 10 patients (Figure 4L). We hypothesized that Prdx2 may play a crucial role in CSC biology. Therefore, we sought to explore the significance of Prdx2 in colon cancer stem cells.

**Table 1:** Case Description and Tumor Features

Patients	Age/Sex	TNM	Tumor size, cm
P1	80/F	T3N0M0	2×3
P2	70/M	T4N0M1	3×4
P3	77/M	T2N0M0	2.5×3
P4	65/F	T3N0M0	2×2
P5	61/M	T4N0M0	4×5
P6	74/M	T4N0M0	4×5
P7	40/M	T4N0M0	3×5
P8	69/M	T2N0M0	3×3
P9	83/M	T3N0M0	4×4
P10	67/F	T3N0M0	3.5×3.5

### Prdx2 deficiency impairs tumor growth *in vivo*

**Figure 5:**
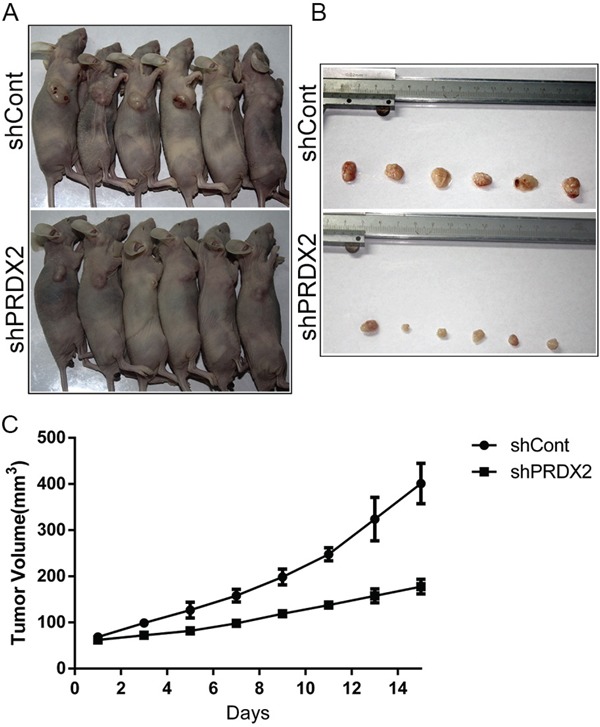
Prdx2 depletion inhibits tumor growth of colon cancer *in vivo* **A-B**. Six mice were placed in each group (shCont and shPrdx2). Images of the mice with tumors and tumors obtained from the mice are presented. **C**. Tumor volume data are represented as the mean value of tumor volume ± the mean standard error.

To evaluate the *in vivo* effects of Prdx2 knockdown, we used a subcutaneous xenotransplant tumor model by injecting the CD133^+^ cells sorted from HCT116-shPrdx2 or HCT116-shCont into female BALB/c-nu mice. The CD133^+^ cells from HCT116-shPrdx2 produced tumors of significantly reduced volume compared with those from HCT116-shCont cells (Figure 5A-5C). This finding indicates that Prdx2 contributes to tumorigenic ability of CSCs in colon cancer.

### Prdx2 activates the Hh/Gli1 signaling pathway in colon CSCs

**Figure 6:**
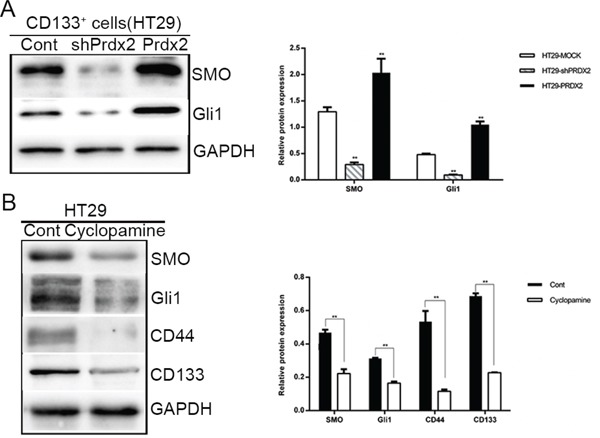
Prdx2 activates the Hedgehog/Gli1 signaling pathway in colon cancer stem cells **A**. The key components of the Hedgehog/Gli1 signaling pathway, SMO and Gli1, were measured by Western blot analysis in CD133^+^ HT29 cells with the knockdown or overexpression of Prdx2. *p< 0.05, **p< 0.01. **B**. SMO, Gli1, CD44, and CD133 protein levels were measured by Western blot analysis in HT29 cell lines treated with cyclopamine (20 μM) for 48 h. *p< 0.05, **p< 0.01.

Hh/Gli1 pathway plays an important role in CSC biology. In our study, Prdx2, SMO, Gli1, and CD133 expression was verified in the colon cancer cell lines. Knockdown or overexpression of Prdx2 markedly reduced or increased, respectively, SMO and Gli1 protein expression in the CD133^+^ cells sorted from the HT29 cells (Figure 6A). When the Hh pathway was inhibited in HT29 cells with the SMO inhibitor cyclopamine, reduced expression of stemness markers, such as CD44 and CD133, was observed (Figure 6B), suggesting that Prdx2 may promote CSC-associated properties in colon cancer via the Hh/Gli1 signaling pathway.

## DISCUSSION

Tumors are currently considered a heterogeneous and hierarchical cellular organization [16]. The redox state plays important roles in cancer cell and cancer stem cell biology [17–19]. CSCs possess unique biological properties, including immortality, tumorigenicity, and resistance to chemical drugs, and they have been identified and isolated in colon cancer [20, 21]. A better understanding of CSC biology is crucial for the development of novel therapies that could effectively and specifically target these cells in colon cancer patients. As is the case for normal stem cells, CSCs are believed to depend on a similar environment, the CSC niche. The redox state modulates the balance between self-renewal and differentiation in CSC niche. The CSC niches are characterized by low levels of ROS, which is critical for maintaining self-renewal and stemness of stem cells [3, 22–24]. While high levels of ROS could effectively shut down self-renewal and induce stem cell differentiation [25–31]. CSCs contain enhanced antioxidative enzyme systems including glutathione (GSH), superoxide dismutases (SOD) and peroxiredoxins compared to their non-tumorigenic progeny, which contributes to lower ROS levels in stem cell niche [3, 5, 32–34]. Prdxs may be a potential target to influence CSC self-renewal and stemness.

Human CD133^+^ (AC133^+^) can be used to identify a population of cells highly enriched for CSCs [15, 20, 35–37]. CD44 and Lgr5 has also been shown to be a marker of CSCs in colon cancer [21, 38]. Nanog is a stem cell transcription factor that is essential for promoting stemness in CRC [39, 40].

Peroxiredoxins (Prdxs) are a family of six isoenzymes and considered to be amongst the most important antioxidant enzymes, known to balance the production of cellular H_2_O_2_ [41–43]. Prdx2 has been reported to be overexpressed in colon carcinomas compared with normal colon tissues [14]. In the present study, we tested the expression of Prdx2 and CD133 in human colon cancer tissues and found a positive correlation between Prdx2 and CD133. Prdx2 expression in stem cells differs in differentiated cells [44, 45]. A recent study revealed that Prdx3 was highly expressed in colon CSCs compared with non-CSCs [46]. In our study we found that Prdx2 was highly expressed in colon CSCs (CD133^+^ cells) compared with non-CSCs (CD133^-^ cells). Prdx2 depletion gives rise to decreased cell proliferation and enhanced apoptosis in colon cancer cells [10]. Prdxs function as a protector of ESC stemness by opposing ROS during neurogenesis and Prdxs depletion leads to differentiation into neurons [47]. Prdx2 depletion attenuated stemness of CSCs in hepatocellular carcinoma [48]. Prdx3 depletion resulted in a smaller percentage of CD133^+^ cells, a significant reduction in sphere formation, and enhanced sensitivity to 5-FU [46]. Prdx4 plays the critical role for reducing oxidative stress in GSCs [49]. Prdx4 inactivation with Piperlongumine may be useful as a novel therapeutic agent for HGG [50, 51]. In our study, knockdown and overexpression of Prdx2 in the colon cancer cell lines SW620, HT29, and HCT116 caused lower and higher CD133^+^ populations, respectively, as well as concomitant decreased and increased transcription and translation levels of CD44, CD133, Lgr5 and Nanog expression, as well as sphere formation. Subcutaneous injection of Prdx2-knockdown cells gave rise to tumors of reduced size in BALB/c-nu mice.

Furthermore, we found that Prdx2 promotes CSC potential via Hh signaling. Hh signaling starts with the secretion of Hh ligand, followed by the secretion of Patched (PTC), transmembrane protein Smoothened (SMO), and several Gli zinc finger transcription factors [39]. Of the three Gli proteins, Gli1 is the final and key output of Hh signaling. Gulino *et al*. found that the Hh/GLi1 pathway played an important role in promoting carcinoma growth, stem cell self-renewal, and metastatic behavior in advanced colon cancers [52]. Human colon CSCs require active Hh/Gli1 signaling for survival and self-renewal [53]. In accordance with those observations, our study showed that knockdown of Prdx2 in CD133^+^ cells sorted from the HT29 cell line inhibited the expression of SMO and GLi1 compared with the control group, which indicates that Prdx2 promotes the properties of colon CSCs via the Hh/Gli1 signaling pathway. In our study, we found that cyclopamine, the best known inhibitor of Hh signaling, suppressed CD133 and CD44 protein levels and inhibited sphere formation in the HT29 cell line, implying that Hh inhibition results in fewer CD133^+^ cells among HT29 cells.

Several anticancer drugs could produce high levels of ROS by depleting GSH or inactiving thioredoxin (Trx), Which has been employed to improve the cytotoxic effects of conventional drugs [54, 55]. Imexon, a prooxidant molecule, depletes intracellular thiols generating oxidative stress and induces apoptosis. Phase I studies is successful in advanced breast and pancreatic tumors, and a phase II study has been carried out [56, 57]. PX-12 (1-methylpropyl 2-imidazolyl disulfide) irreversibly inactivates Trx-1 and the antitumor activity can be synergistically enhanced after combination of PX-12 with 5-FU in HCC cells [58]. Pharmacological depletion of Prdx2 may be a potential tragedy to improve the effects of chemotherapeutic drugs in gastrointestinal cancer.

In our study, we reported that Prdx2 acts as a promotor of CSC properties in colon cancer. Mechanistically, we propose that the Hh signaling pathway may play an important role in the association between Prdx2 and CSC-associated properties. The data we present suggest that depletion of Prdx2 or Hh/Gli signaling would be beneficial for suppressing CSCs in the treatment of colon carcinoma. Of course, more studies should be conducted in additional cancerous and control tissues as well as primary cells.

## MATERIALS AND METHODS

### Cell culture and reagents

The human colon cancer cell lines SW620, HT29, and HCT116 were obtained from the Shanghai Cell Bank at the Chinese Academy of Sciences (Shanghai, China). The cell lines were maintained in 1640 medium (Gibco, Grand Island, NY, USA) containing 10% fetal bovine serum (PAN, Aidenbach, Bavaria, Germany) at 37°C in a humidified incubator.

Antibodies against Prdx2 and Gli1 were purchased from Abcam plc (UK). Antibodies against Nanog and CD44 were purchased from Cell Signaling Technology (Danvers, MA, USA). Antibodies against CD133 were purchased from Proteintech (USA). Fluorescently-tagged antibodies against CD44 and CD133 were purchased from Miltenyi Biotec (Germany).

### Magnetic-activated cell sorting and flow cytometry

Human colon cancer cell lines HT29, HCT-116, and SW620 were dissociated into single-cell suspensions, washed with phosphate-buffered saline (PBS), and stained with an AC133/CD133-PE antibody (Miltenyi Biotec). Magnetic cell separation was performed on a column-free cell isolation platform using an AC133/CD133-PE antibody (Miltenyi Biotec) and the EasySep™ Human PE Positive Selection Kit (STEMCELL Technology, USA). After magnetic sorting, both the CD133^+^ and CD133^-^ cell populations were assessed by flow cytometry using a CD133-PE antibody (Miltenyi Biotec). Apoptosis was measured by labeling cells with 7-AAD-PE and Annexin V-PE (BD Biosciences, CA), as recommended by the manufacturer.

### Transfection and stable cell line construction

Lentiviral constructs expressing Prdx2 shRNA (Prdx2-shRNA-LV) were purchased from Genechem (Shanghai, China). The Prdx2 shRNA vector sequence was as follows: forward 5’-TCC TCT TTA TCA TCG ATG GCA ACT CGA GTT GCC ATC GAT GAT AAA GAG GTT TTT TC-3’; reverse 3’-TCG AGA AAA AAC CTC TTT ATC ATC GAT GGC AAC TCG AGT TGC CAT CGA TGA TAA AGA GGA-5’. Prdx2-shRNA-LV was transduced into cells at a multiplicity of infection (MOI) of 60 using polybrene (10μg/ml) and Enhanced Infection Solution (Genechem, China). At the same time, a non-target negative control virus GFP-LV was transduced into cells for control. For Prdx2 overexpression, the cells were transfected with lentiviral particles expressing Prdx2 or GFP as the negative control. Infected cells were selected with media containing 5 μg/ml puromycin.

### RNA extraction and qRT-PCR analysis

Cellular RNA was extracted from cells using the RNAiso plus reagent (Takara). RNA (1 μg) was reverse-transcribed with the PrimeScript™ RT reagent Kit and gDNA Eraser (Takara). All the reactions were performed in triplicate, and the *B2M* gene was used as the internal control. The primer sequences used in this study are provided in Table 2. Using the comparative threshold cycle (cq) method, the relative quantification of gene expression was calculated as the ratio of CSCs to non-CSCs after normalization against B2M for each sample.

**Table 2:** QRT-PCR Primer Sequences Used in the Current Study

Gene	accession number	Primer sequences		
		Forward	Reverse	Product size (bp)
Prdx2	NM_005809	GCTGGGCTGTGAAGTGCTGG	ACGCCGTAATCCTCAGACAAGC	150
NANOG	NM_024865	CCTATGCCTGTGATTTGTGGG	TTGCCTTTGGGACTGGTGG	155
EPCAM	NM_002354	CAAGGACACTGAAATAACCTGCTC	CTCCTTCTGAAGTGCAGTCCG	124
CD44	NM_000610	ATCATCTTGGCATCCCTCTTG	CACCATTTCCTGAGACTTGCTG	177
CD133	NM_006017	ACAATCCTGTTATGACAAGCCCA	GGAAAGTCCTTGTAGACCCAGAAA	126
B2M	NM_004048	CTCTTTCTGGCCTGGAGGCTAT	AGTCAACTTCAATGTCGGATGGAT	135

### Protein isolation and western blot

Cells were rinsed with PBS and then lysed in Lysis Buffer. The crude lysate was centrifuged at 12,000 rpm for 20 min, and the clarified cell extract was used for immunoblotting. Proteins were separated by sodium dodecyl sulfate polyacrylamide gel electrophoresis (SDS-PAGE), transferred onto polyvinylidenefluoride (PVDF) membranes (Immobilon-P, Millipore, Germany), blocked with 5% skim milk in TBST (20 mM Tris-HCl, 150 mM NaCl, 0.1% Tween 20), and blotted with the appropriate primary and secondary antibodies. The antigen-antibody complexes were detected by chemiluminescence (Millipore, Germany).

### Sphere formation culture

For sphere formation, cells were seeded in ultra-low-attachment 6-well plates (Corning, USA) at a density of 10^4^ cells/well in serum-free medium (DMEM/F12) containing epidermal growth factor (20 ng/ml, Peprotech), basic fibroblast growth factor (20 ng/ml, Peprotech), and B-27 (0.4%, Invitrogen). The numbers of spheroids were counted 14 days later.

### Clonogenic cell survival assay

Cells (1×10^5^) were plated into 24-well plates. After overnight incubation, cells were treated with 5-FU followed by 8 days of incubation. The colonies were fixed and stained with 0.5% crystal violet, washed, dried and imaged. Crystal violet was resolved from colonies by methanol and measured at 540 nm. Based on the absorbance at 540 nm, survival curves were expressed as a percentage ± SD relative to DMSO-treated control from three independent experiments [59].

### Immunohistochemistry of tissue samples

Tissue samples were fixed in 4% paraformaldehyde, embedded in paraffin, and cut into 5-μm-thick sections. Sections were deparaffinized in xylene and rehydrated with graded ethanol. Antigen retrieval was performed by boiling the samples in sodium citrate buffer (0.01 mM). Endogenous peroxidases were inactivated with 3% H_2_O_2_, followed by incubation with goat serum for 20 min at 37°C, with the primary antibody overnight at 4°C, and with the secondary antibody for 20 min at 37°C in a humidified chamber. Peroxidases bound to the antibody complex were visualized by treatment with a 3,3-diaminobenzidine chromogenic substrate solution. Immunolabeled sections were dehydrated with graded ethanol and defatted in xylenes. The sections were then visualized using an Olympus BX51 microscope (Olympus, Japan) under bright-field illumination, and images were acquired with an Olympus DP70 camera (Olympus, Japan). Images were processed and Average Integrated Optical Density (AIOD) were obtained from 10 random 200x microscopic fields with image-pro plus version 6.0(Media Cybernetics, Bethesda, MD, USA). The study involving human samples was approved by the Ethics Committee of the First Affiliated Hospital of Chongqing Medical University (Chongqing, China). Informed consent has been obtained for the collection of tissues and subsequent analysis.

### Evaluation of tumorigenicity

Tumorigenicity was determined by subcutaneously injecting 5×10^4^ HCT116-shCont or HCT116-shPrdx2 CD133^+^ cells into the flanks of 6-week-old female BALB/c-nu mice (Animal Center of Chongqing Medical University, China). All studies involving animals were approved by the Ethics Committee of Chongqing Medical University. The tumor size was measured every 2 days using a caliper. The tumor volume (V= l*w^2^/2) was calculated by measuring the length (l) and width (w). Mice were euthanized 15 days after cell injection.

### Statistical analysis

Statistical analysis was performed using SPSS software, version 21.0 (SPSS, Chicago, IL, USA) and GraphPad Prism Version 6.02 (GraphPad Software, La Jolla, CA, USA). Student's *t*-test was used to evaluate the significance of the observed differences between any two groups of data, and one-way analysis of variance (ANOVA) was used to evaluate the significance of differences between multiple comparisons. Correlation analysis was performed using the Pearson method. Data are represented as the mean ± the standard deviation of at least three independent experiments. The value of *P* < 0.05 was considered significant.
